# Parameters in Canines After Cesarean Sections

**DOI:** 10.3389/fvets.2022.886691

**Published:** 2022-06-16

**Authors:** Theresa Conze, Kathrin Büttner, Axel Wehrend

**Affiliations:** ^1^Clinic for Obstetrics, Gynecology and Andrology of Large and Small Animals With Ambulatory Service, Faculty of Veterinary Medicine, Justus-Liebig-University Giessen, Giessen, Germany; ^2^Units for Biomathematics and Data Processing, Faculty of Veterinary Medicine, Justus-Liebig-University Giessen, Giessen, Germany

**Keywords:** parturition, cesarean section, canine, dogs, fertility

## Abstract

This study evaluated fertility in canines after cesarean section and compared it with natural parturition. Parameters, such as the time of the next heat after the first parturition or cesarean section, the heat which was used for another breeding attempt, whether it was successful, the number of puppies that were born, and the necessity of another cesarean section were examined. The study relied on questioning patient owners at a University clinic. A Google online form was also used. Information for 261 dogs from different breed groups was included, of which 119 bitches were in the cesarean section group, and 142 were in the natural parturition group. In total, 93 ± 2.7% [LSMeans ± standard error (SE)] and 91.12 ± 3% (LSMeans ± SE) of the bitches became pregnant after cesarean section and natural parturition at the first breeding attempt. There was no significant effect on the breed group or whether the bitch had undergone a cesarean section before (*p* = 0.8 and *p* = 0.63). Bitches, which underwent a cesarean section, were more likely to have further cesarean sections performed (*p* < 0.001). However, neither the breed groups (*p* = 0.17), whether the bitch had undergone a cesarean section (*p* = 0.59), nor the number of previous parities had any effect on the number of puppies born (*p* = 0.95). The breed group bulldogs had a high proportion of cesarean sections. Only 42.11% of the bulldogs had a natural parturition as the first included parturition and only 31% gave birth naturally thereafter.

## Introduction

With the increasing popularity of certain breeds, the incidence of cesarean sections is rising. Unlike other surgeries, cesarean delivery may be both scheduled and predictable intervention (1–4), as well as an emergency surgery (5). Dystocia occurs in ~5–16% of all bitches during parturition (6), of which ~60–80% result in a cesarean section (7, 8). Especially brachycephalic breeds are known to have a high risk of cesarean sections and breeds, such as the Scottish Terrier (6, 9, 10).

With the rising incidence of cesarean sections and the increasing desire for planned cesarean sections (11, 12), the fertility and the question if one can expect a natural delivery after a cesarean section are important aspects. Although data from human medicine show varying results (13–16), little is known about the fertility after cesarean sections in dogs (17). In human medicine, the fertility rates show results as no reduction in fertility up to 17% (13–15). In their retrospective study, Conze et al. (17) determined a fertility rate of 100% in 55 bitches, which became pregnant in the 2 years following a cesarean section. No information was included about the breeds, the need for another cesarean section in subsequent pregnancies, and a possible reduction in the number of puppies that were born. Seyrek-Intas et al. (18) performed a unilateral cornuectomy of the uterus of 18 healthy bitches. Twelve bitches were mated during the first postoperative estrus. Of these, nine delivered puppies. This indicates high fertility, but in the study by Conze et al. (17), only a small population of bitches was examined, which limits the strength of their conclusions. These numbers have to be compared to normal pregnancy rates, which are between 78 and 97% (19–21).

Only a few studies have looked into the fertility parameters of certain breeds (22, 23). Cafaratti et al. (22) examined clinical signs during estrous and mating in the Dogo Argentino and the risk of dystocia and cesarean section. Linde Forsberg and Persson (23) performed a survey on boxers to investigate parameters, such as the estrus interval and the occurrence of dystocia. There is, however, a definite lack in the literature examining and comparing fertility parameters after cesarean section in different breed groups.

## Materials and Methods

### Gathering of Information

Two methods were used for gathering information. Firstly, breeders who were clients of the Clinic for Obstetrics, Gynecology and Andrology of Large and Small Animals with Ambulatory Service, Faculty of Veterinary Medicine were interviewed. They were asked if they would voluntarily provide information about their breeding bitches during registration. No exclusions were made regarding breeds. Bitches having experienced at least two parturitions were included. In the cesarean section group, the first cesarean section to be included had to be an emergency cesarean section. Breeders were interviewed using a standardized questionnaire. A Google form with the same questions was used as a second data collection method. It was sent to clients, who then forwarded it to other breeders. The link to the questionnaire was also accessible *via* the Verein Deutscher Hundezucht (VDH) website and was distributed *via* social media. The same inclusion and exclusion criteria were applied to the Google form. The data were collected between 2017 and 2019.

### Parameters

Breed, age, weight, and the physiological inter-estrous interval, which was defined as the time between the non-pregnant bitch's heats, were collected for cesarean section (G1) and natural parturition group (G2). Furthermore, in the cesarean section group following parameters were included (G1): the indication for the cesarean section was assessed. The number of puppies born by cesarean section and the time of the first heat after cesarean section were included. The interval between cesarean section and the following breeding, counted as heat numbers, as well as the outcome of the breeding, was examined. The following parameters were collected in the natural parturition group (G2): questions regarding the number of puppies born in the first included parturition and the time of the first heat after parturition were asked. The interval between parturition and the following breeding, counted as heat numbers, was included, as well as the outcome of the breeding.

If a bitch from G1 or G2 became pregnant again, the breeders were asked whether the bitch was able to give birth naturally and how many puppies were born. If the bitch had undergone a cesarean section, the breeders were asked to state the indication.

### Statistical Analysis

The statistical software package SAS 9.4 (24) was used for statistical analysis. Mixed linear models were applied to normally distributed data using the MIXED procedure. The parameters for this analysis were as follows: the time of first heat after cesarean section, difference of puppies born between G1 and G2, and the number of the first heat used for breeding after a cesarean section or natural parturition. Generalized mixed linear models were applied to the analysis of binomial data, such as the variable of a positive pregnancy at the next breeding attempt (0 = no, 1 = yes) and the variable following parturition (0 = no natural parturition, 1 = natural parturition) using the generalized mixed linear models (GLIMMIX) procedure. Due to the fact that Terriers always got pregnant, the model could not represent proper estimates for this parameter. Therefore, it was calculated again without the breed group terrier.

The Akaike's information criteria corrected (AICC) (25) and the Bayesian information criteria (BIC) (26) were used to compare the different models. The model with the smallest AICC and BIC values was chosen. In general, the fixed effects breed group, type of parturition (G1: cesarean section, G2: natural parturition) and the covariate parities (number of parturition which was included as first parturition in the study) were added stepwise to the models. Interactions between the fixed effects were also tested, but none of the interactions was significant. Therefore, the interactions were removed from the model. The pairwise comparisons of the least square means were adjusted by the Bonferroni-Holm correction (27).

The results for the descriptive statistics are illustrated as mean ± standard deviation (SD). The results from the statistical models are illustrated as least square means (LSMeans) ± standard error (SE).

## Results

### Breeds, Age, Weight, Number of Parturition, and Indication for Cesarean Section

Altogether, 261 bitches were included in the study, of which 119 bitches were in G1 (45.59%) and 142 were in G2 (54.41%). The arithmetic mean age ± SD of bitches at the time the cesarean section was performed and was 3.35 ± 1.15 (1–6 years) in G1 whereas the arithmetic mean age ± SD of the bitches in G2 was 2.69 ± 0.74 years (1.5–7 years) at parturition. The bitches in G1 had a mean weight of 24.93 ± 13.45 kg (2.2–63 kg), whereas the mean weight in G2 was 23.54 ± 12.12 kg (3.3–68 kg). The following breed groups where included: bulldogs (*n* = 19), herding dogs (*n* = 38), molossers (*n* = 41), retrievers (*n* = 60), shepherds (*n* = 17), and terriers (*n* = 28). In total, 58 dogs could not be assigned to the groups mentioned (Schnauzer: *n* = 7; Sennenhund: *n* = 7; European and Asian Spitz: *n* = 7, cocker: *n* = 7, Dalmatiner: *n* = 7, Pudel: *n* = 6, Petit Brabancon: *n* = 3, pug: *n* = 3, Coton de Tulear: *n* = 2, Shih Tzu: *n* = 2, Chihuahua, Dachshound, Weimaraner, Whippet, Papillon, Pumi, and Xoloitzcuintle standard). They were placed in the group “others” (Table 1). The breeders stated the following indications for cesarean section: uterine inertia (*n* = 48), malpresentation of the puppy (20), fetomaternal disproportion (*n* = 15), dead fetus (11), singleton (*n* = 5), green vaginal discharge, (2) brown vaginal discharge before the birth of the first puppy (*n* = 1) and uterine convulsion (1), wedged puppies (1), and maternal circulatory problems (1).

**Table 1:** Included breed groups.

**Number**	**Breed**	**Number of**	**Median age at first**
	**group**	**bitches**	**included parturition and first**
		**included (*n*)**	**quartil and third quartil (years)**
1	Bulldogs	19	2 / 2 / 2.75
2	Herding dogs	38	3 / 2.5 / 3.37
3	Molossers	41	3 / 2.5 / 3
4	Retrievers	60	3 / 2.5 / 4
5	Shepherds	17	3 / 2.5 / 3
6	Terriers	28	2.5 / 2 / 3
7	“Others”	58	3/ 2.05 / 3

### Distribution of First Parturition Depending on Breeds

Of the bulldogs, 57.89% (*n* = 11) had undergone a cesarean section during the first parturition included, and 42.11% (*n* = 8) gave birth naturally. Only 42.86% (*n* = 12) of the terriers had given birth naturally as compared to the herding dogs, molossers, and retrievers, where 63.16% (*n* = 24), 60.98% (*n* = 25), and 56.67% (*n* = 34) were able to give birth naturally.

### Physiological Inter-estrous Interval

The physiological inter-estrous interval in G1 was 6.56 ± 1.88 months (1–13 months), whereas the physiological inter-estrous interval was 7.21 ± 1.84 months (3.5–13 months) in G2.

### Number of Puppies Born

Altogether 1,651 puppies were born in the first parturition. In total, 727 puppies were born in G1 and 924 puppies were born in G2. In G1 6.11 ± 3.18 (1–13) puppies were born per bitch in the first documented cesarean section, whereas 6.51 ± 2.47 (1–12) puppies were born in G2 in the first parturition per bitch.

### Following Heat

The LSMean for the first heat after cesarean section (G1) was 6.75 ± 0.19 months and 6.81 ± 0.19 months after natural parturition (G2). Altogether bitches showed their first heat after 1–13 months and 1–14 months after the previous parturition in G1 and G2, respectively. The type of parturition and the covariant parities had no significant effect on the first heat after cesarean section (*p* = 0.8 and *p* = 0.35). The breed group had a significant effect on the first heat after cesarean section (*p* < 0.05). There was a significant difference between the breed group molossers (LSMean 7.48 ± 0.31 months) and the group “others” (LSMean 6.22 ± 0.62 months; *p* < 0.05) and between the breed group retrievers (LSMean 7.65 ± 0.26 months) and the group “others” (LSMean 6.22 ± 0.62 months; *p* < 0.05). Furthermore, retrievers came into heat significantly later post-parturition (LSMean 7.65 ± 0.26 months) than the breed group terriers (LSMean 6.22 ± 0.26 months; *p* < 0.05).

### Following Breeding

The LSMean for the interval between cesarean section and following breeding in G1 and parturition and following breeding in G2 counted as heat numbers was 2.3 ± 0.11 in G1 and 2.42 ± 0.1 in G2. The first heat was used in 19.3% (*n* = 23) of the bitches in G1 and 14.08% (*n* = 20) in G2, whereas 47.90% (*n* = 57) of the bitches in G1 and 47.89% (*n* = 68) of the bitches in G2 were bred during the second heat after the previous cesarean section or parturition. The third heat was used for breeding in 21.85% (*n* = 26) and 23.24% (*n* = 33) of bitches in G1 and G2. Looking into the fbreed distribution, none of the bulldog bitches was mated during the first heat, whereas 35.29% (*n* = 6) of shepherds were mated during their first heat (Table 2).

**Table 2:** Distribution (%) of mated bitches depending on the used heat number and the breed group after a previous parturition.

	**Used heat number for breeding**							
**Breed group**	**1**	**2**	**3**	**4**	**5**	**6**	**7**	**9**
Bulldogs	0.00	52.63	31.58	5.26	10.53	0.00	0.00	0.00
Herding Dogs	15.79	31.58	31.58	13.16	2.63	0.00	0.00	2.63
Molossers	29.27	46.34	17.07	4.88	0.00	0.00	2.44	0.00
Retrievers	18.33	50.00	23.33	3.33	5.00	0.00	0.00	0.00
Shepherds	35.29	47.06	17.65	0.00	0.00	0.00	0.00	0.00
Terriers	17.86	53.57	21.43	3.57	3.57	0.00	0.00	0.00
“Others”	5.17	53.45	18.97	13.79	6.90	1.72	0.00	0.00
Sum	16.48	47.89	22.61	7.28	4.21	0.77	0.34	0.38

The type of parturition had no significant effect on the first used heat after parturition (*p* = 0.42). The fbreed group did have a significant effect on the first used heat after parturition (*p* < 0.05). However, there are no longer any significant effects in the multiple pairwise comparisons for the adjusted values of *p*. Due to the many comparisons between the individual breeds, the alfa level is greatly reduced so that no significant values remain after the adjustment. The covariant parities had no significant effect on the dependent variable (*p* = 0.58).

### Following Pregnancy

Altogether 241 bitches became pregnant in the first breeding attempt. Within G1, 112 bitches became pregnant at the first breeding attempt, whereas 129 bitches became pregnant at the first breeding attempt in G2. The LSMean of bitches getting pregnant in the next heat used was 0.93 ± 0.027 in G1 and 0.91 ± 0.03 in G2. With probability values of 93 ± 2.7% and 91.12 ± 3%, bitches became pregnant in G1 and G2 at the first breeding attempt after the previous parturition. There was no significant effect of the fixed effects on breed group and cesarean section (*p* = 0.8 and *p* = 0.63) and the covariant parities (*p* = 0.33).

The pregnancy rate was found to be between 86.5% (*n* = 16) (bulldogs) and 100% (*n* = 28) (terriers).

### Following Parturition

Of the original 261 bitches, 246 (94.25%) gave birth again. In G1, 52 of the 112 bitches had undergone a cesarean section, while 60 whelped naturally. As the following indications were stated by the owners: uterine inertia (*n* = 23), fetomaternal disproportion (*n* = 6), no signs of parturition (*n* = 3), dead puppy (*n* = 2), singleton (*n* = 2), weak heartbeats of the offspring (2), physiologically the bitch was not able to give birth naturally (*n* = 1), brown discharge (*n* = 1), the cervix was not dilated (1), planned cesarean section (1), and uterine rupture (1). Similarly, 17 (12.69%) of the 134 bitches in G2 had undergone a cesarean section while 117 (87.31%) whelped naturally. The breeders stated the following indications: fetomaternal disproportion (3) and uterine inertia (3), planned cesarean section due to myositis (1) and insufficient amniotic fluid (1), and singleton (1).

**Figure 1:**
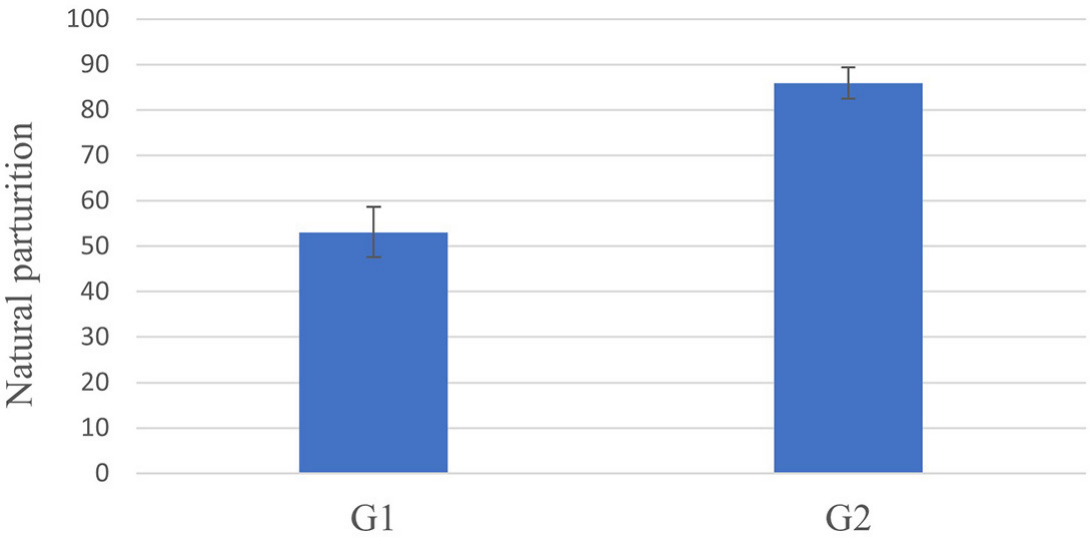
Estimated frequencies [LSMeans ± standard error (SE)] for natural parturition depending on G1 and G2. Animals in G2 had experienced natural parturition more often with 85.94 ± 3.43% as compared to the animals in G1 with 53.08 ± 5.51% (*p* < 0.05).

The breed group and the type of parturition both had a significant effect on natural parturition (*p* < 0.05). Regarding the cesarean section, animals in G2 had experienced natural parturition more often with 85.94 ± 3.43% (LSMeans ± SE) as compared to the animals in G1 with 53.08 ± 5.51% (LSMeans ± SE; *p* < 0.05; Figure 1).

**Figure 2:**
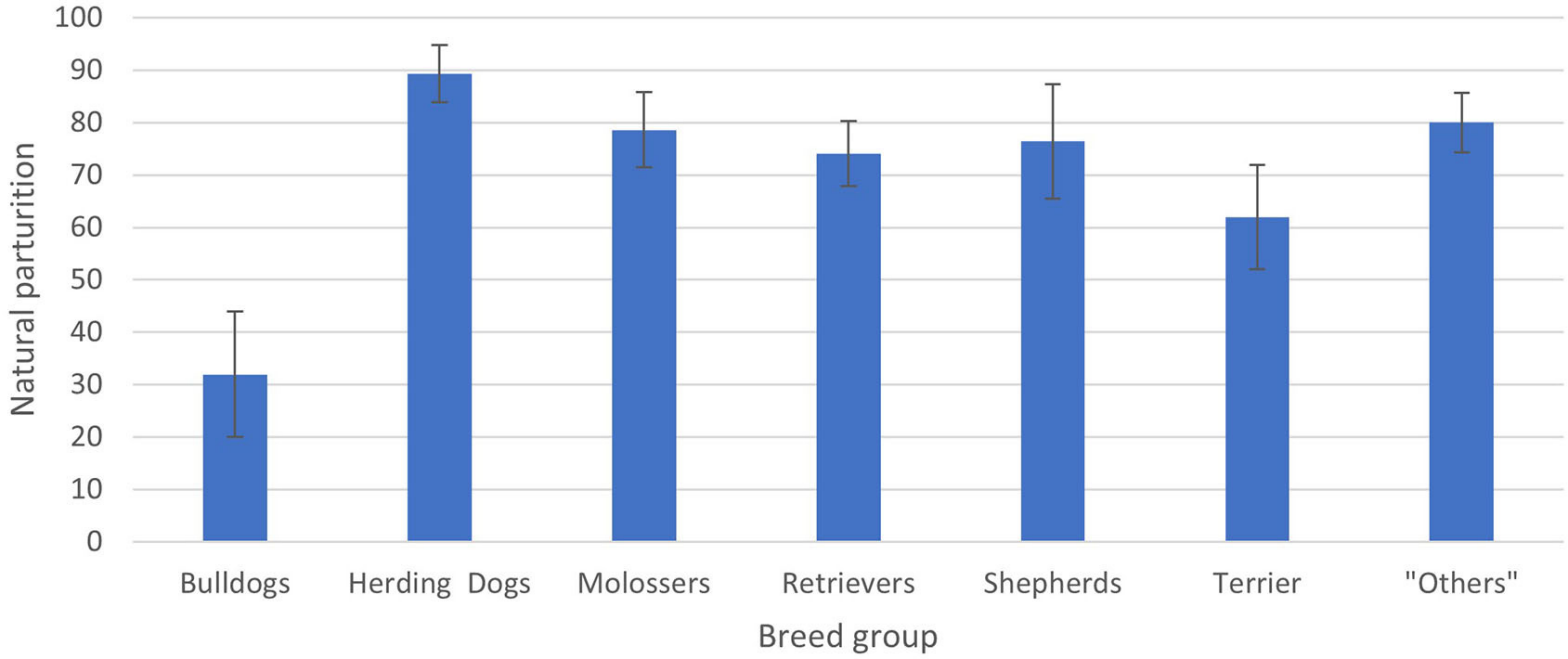
Estimated frequencies [LSMeans ± standard error (SE)] for natural parturition depending on breed groups in case of pregnancy after a previous parturition. Significant differences could be obtained for the comparisons between breed group 1 (bulldogs) (31.95 ± 12.00%) and 2 (herding dogs) (89.30 ± 5.44%; *p* = 0.0079) and between breed group 1 (bulldogs) and 6 (others) (80.07 ± 5.66%; *p* = 0.0285). All other comparisons showed no significant differences (*p* > 0.05).

There was a significant difference between bulldogs, where only 31.95 ± 12% (LSMeans ± standard error) had experienced natural parturition compared to herding dogs, where 89.30 ± 5.44% (LSMeans ± standard error) were able to give birth naturally (*p* = 0.0079) as well as between bulldogs (31.95 ± 12%) and “others” (80.07 ± 5.66%) (*p* = 0.0285) (Figure 2). All other comparisons showed no significant differences (*p* > 0.05).

### Puppies Born During the Following Parturition

Altogether 1,583 puppies were born in the parturition following the first recorded parturition. In G1, 671 and in G2, 912 puppies were born. Each bitch birthed a mean of 6.04 ± 2.86 puppies in G1 (1–13), and in G2, each bitch birthed a mean of 6.8 ± 2.59 puppies (1–15).

The LSMean of the difference in the number of puppies born in G1 was 0.17 ± 0.3 and 0.39 ± 0.28 in G2. The breed group and the type of parturition had no significant effect on the difference in the number of puppies born (*p* = 0.17 and *p* = 0.59). The covariant number of parities had no significant effect on the dependent variable “difference in number of puppies born” (*p* = 0.95).

## Discussion

In this study, 261 bitches were included, of which 45.59% (*n* = 119) had a cesarean section and 54.41% (*n* = 142) gave birth naturally. Even if the homogeneity of the groups was not statistically calculated, the bitches in both groups showed similar body masses (G1: 24.93 ± 13.45 kg vs. G2: 23.54 ± 12.12 kg) and similar ages (G1: 3.35 ± 1.15 years vs. G2: 2.67 ± 0.74). The comparison of these groups, therefore, reveals some interesting fertility parameters.

Forsberg and Persson (23) found that the need for veterinary treatment and cesarean sections increased with the bitches' age. Bitches over 4 years old were at an increased risk. In this study, the mean age in G1 was 3.35 ± 1.15 years. A possible explanation could be that only boxers were included in the study of Forsberg and Persson (23), whereas this study examined different breeds.

There was no difference in the first heat's time occurrence regardless of whether the bitches had undergone a cesarean section or experienced natural birth (G1: 6.75 ± 0.19 vs. G2: 6.81 ± 0.19 months). There were differences in the time occurrence of the first heat after parturition between the breed groups, which matches the findings of other studies (28). Thus, cesarean sections do not seem to affect the resumption of the ovarian cyclicity. However, breed group does seem to have an effect on the resumption of the ovarian cyclicity and, therefore, impacts the time of heat occurrence. The first heat after parturition was used for mating in 19.3% (*n* = 23) (G1) and 14.08% (*n* = 20) (G2) of the bitches, whereas the majority of the bitches were bred in the second heat (G1: 47.90% vs. G2: 47.89%). It appears that having a cesarean section does not affect the owner's decision as to when to breed the bitch again. In human medicine, it is often recommended that women should only become pregnant again after a certain amount of time if they had a cesarean section (29). Short inter-pregnancy intervals in women are associated with a higher risk of complications during pregnancy and birth (29–31). Corresponding recommendations for dog breeders are not known to the authors. However, considering that dogs have a placenta endochorialis as compared to the placenta hemochorialis in women, the risk factors apparent in human medicine might not play a role in dogs. A factor well known in humans, that might play a role in dogs, is an increased risk of uterine rupture after a cesarean section (29). Nevertheless, only one dog owner reported uterine rupture as an indication for cesarean section.

With a probability of 93 ± 2.7% and 91.12 ± 3%, bitches became pregnant again in G1 and G2 during the first breeding attempt. There was no significant effect on the bitches' breed or whether they had experienced a cesarean before, on the likelihood of this pregnancy (*p* = 0.8 and *p* = 0.63, respectively). However, whether there is an impact on pregnancy success or incidence of complications during pregnancy from using the first or the second heat after the cesarean section remains an open question.

There was no significant difference between breeds (*p* = 0.63) regarding the pregnancy rate. In human medicine, on the other hand, studies indicate a reduction of fertility between 0 and 27% (13–15, 32). This seems to play a subordinate role in dog breeding. In the study by Conze et al. (17) pregnancy rates of 100% were found in 55 bitches within the 2 years following parturition. However, there was a lack of more precise data as to how often the bitches were bred again before getting pregnant. In this study, only the first breeding attempt was used for statistical calculation. In a study by Seyrek-Intas (18), 9 out of 12 bitches that had previously undergone a unilateral cornuectomy were whelped. It has to be taken into consideration that the cornuectomy was performed on healthy dogs. Although one might have expected reduced fertility in bitches with dystocia as compared to healthy bitches, which underwent surgery, this was not confirmed by the study.

Taking a closer look on breed-specific patterns of the first included parturition, 57.89% (*n* = 11) of the bulldogs had a cesarean section, and only 42.11% (*n* = 8) gave birth naturally. A similar proportion can be found in terriers, where only 42.86% (*n* = 12) experienced natural parturition. Therefore, bulldogs and terriers had lower rates of natural parturition as compared to the other breeds: herding dogs, molossers, and retrievers (63.16, 60.98, and 56.67% respectively). This matches the findings of Bergstrom et al. (6) who found that some breeds were at an increased risk of dystocia. Interestingly, they found that the Scottish Terrier is also at a higher risk for dystocia and cesarean sections, which might be in line with the findings of this study regarding the terriers in the first included parturition. However, according to Bergstrom et al. (6), Yorkshire Terriers were not found to be at a high risk for cesarean sections. This suggests that the breeds in the breed groups should be considered individually in subsequent studies. Eneroth et al. (9) reported that the frequency of cesarean sections in French Bulldogs was 43%, which matches the findings of this study.

Altogether, bitches that underwent a cesarean section had a higher likelihood of another cesarean section as compared to bitches, which experienced a first natural parturition. Only 53.08 ± 5.51% (*n* = 60) of the bitches whelped naturally after a cesarean section as compared to 85.94 ± 3.43% (*n* = 117) of the bitches after a natural parturition. The breed group also had a significant effect on the following parturition (*p* < 0.05). A significant difference was recorded between bulldogs, where only 31.95 ± 12% bitches gave birth naturally as compared to the herding dogs, with 89.30 ± 5.44% natural parturition (*p* = 0.0079) as the following parturition. Taking this into account, a future study could evaluate these differences according to individual breeds and not only breed groups. Brachycephalic breeds, in particular, are known to have a high rate of cesarean sections (33).

Furthermore, the question arises whether the subsequent cesarean section had the same indication as the first. Of the 119 bitches in G1, 48 bitches (40.34%) had undergone a cesarean section due to uterine inertia and 20 bitches (16.8%) due to malpresentation of the puppy. In total, 52 of these bitches underwent another cesarean section, where 23 (44.23%) cesarean sections were performed due to uterine inertia. This might indicate that, at least in some bitches, the subsequent cesarean section had the same indication as the first. However, it has to be taken into consideration that the indication for cesarean section was given by an anonymous questionnaire completed by the breeders, so some of the indications may be imprecise. To confirm the result, a retrospective study would be interesting. Never less, our findings match those of Linde Forsberg and Persson (23), who found primary uterine inertia (60%) and malpresentation of the fetus (26%) to be the most common reasons for dystocia.

A mean of 6.11 ± 3.18 (1–13) puppies was born per bitch after the first documented cesarean section (G1) and 6.04 ± 2.86 puppies during the following parturition. Similarly, 6.51 ± 2.47 (1–12) puppies were born in G2 during the first documented parturition per bitch and 6.8 ± 2.59 puppies in the following parturition. This matches the finding of Forsberg and Persson (23). However, the size of the litter is very dependent on the breed, and, as a result, no conclusion can be drawn with regards to the effects of cesareans on litter size. However, neither the breed group (*p* = 0.17) nor whether the bitch had a prior cesarean section (*p* = 0.59) nor the number of previous parturitions had an effect on the number of puppies born (*p* = 0.95). Overall, a prior cesarean section does not reduce the number of puppies born in the following breeding.

## Conclusion

A prior cesarean section appears to have no negative effect on fertility and the number of puppies born in the following breeding. Nevertheless, dog owners must be aware that the risk of a second cesarean section is high. The breeder should, therefore, consider carefully breeding dogs that underwent cesarean section before, as they seem to have a high risk for dystocia. Our data can support counseling by veterinarians regarding the issue, addressing important ethical aspects.

## Data Availability Statement

The raw data supporting the conclusions of this article will be made available by the authors, without undue reservation.

## Author Contributions

KB did the statistical analysis. AW is the head of the study. All authors contributed to the article and approved the submitted version.

## Conflict of Interest

The authors declare that the research was conducted in the absence of any commercial or financial relationships that could be construed as a potential conflict of interest.

## Publisher's Note

All claims expressed in this article are solely those of the authors and do not necessarily represent those of their affiliated organizations, or those of the publisher, the editors and the reviewers. Any product that may be evaluated in this article, or claim that may be made by its manufacturer, is not guaranteed or endorsed by the publisher.
